# Epidemiological Changes in Acute Febrile Diseases after the COVID-19 Pandemic in Thailand

**DOI:** 10.4269/ajtmh.24-0017

**Published:** 2024-11-19

**Authors:** Rapeepun Prasertbun, Hirotake Mori, Yoshiro Hadano, Aongart Mahittikorn, Rapeephan R. Maude, Toshio Naito

**Affiliations:** ^1^ Department of General Medicine, Juntendo University Faculty of Medicine, Tokyo, Japan;; ^2^ Department of Protozoology, Faculty of Tropical Medicine, Mahidol University, Bangkok, Thailand;; ^3^ Division of Infection Control and Prevention, Shimane University Hospital, Izumo, Japan;; ^4^ Division of Infectious Diseases, Department of Medicine, Faculty of Medicine, Ramathibodi Hospital, Bangkok, Thailand;; ^5^ Department of Epidemiology, Mahidol-Oxford Tropical Medicine Research Unit (MORU), Faculty of Tropical Medicine, Mahidol University, Bangkok, Thailand

## Abstract

Acute undifferentiated febrile illnesses (AUFIs) are short-duration infectious diseases with nonspecific symptoms. In Thailand, common AUFIs include dengue, malaria, leptospirosis, scrub typhus, and typhoid fever. This study aimed to determine the case numbers of AUFI etiologies in Thailand before coronavirus disease 2019 (COVID-19) (phase 1 from January 2018 to February 2020) and during the COVID-19 pandemic with preventive measures (phase 2 from March 2020 to April 2022), and the loosening of the preventive measures (phase 3 from May 2022 to December 2022). We used Thailand’s national database from 2018 to 2022 to determine the case numbers of AUFIs and geographic heat maps to identify endemic areas in Thailand. The case numbers of malaria, dengue, leptospirosis, typhoid, and scrub typhus significantly decreased during phase 2 (preventive measures) (*P* = 0.02), and cases of malaria and leptospirosis increased during phase 3 (loosened preventive measures) (*P* = 0.01). In 2022, malaria and leptospirosis increased by 39% and 48%, respectively, compared with the previous year. Malaria increased in western Thailand along the border between Thailand and Myanmar, where malaria preventive measures were insufficient, whereas leptospirosis increased in northern Thailand. The epidemiology of acute febrile diseases changes significantly depending on the global epidemic of infectious diseases such as COVID-19 and the implementation of preventive measures, such as face masks, hand hygiene, social distancing, and stay-at-home and lockdown measures.

## INTRODUCTION

Coronavirus disease 2019 (COVID-19) was first reported in December 2019 and evolved into a pandemic with more than 75 million cases and 1.6 million deaths.^1^ From January 12, 2021 to December 3, 2022, the Ministry of Public Health reported that 4,658,836 people were infected with COVID-19 and 32,949 people had died. The number of daily COVID-19 cases remained below 2,000 from January to November 2020. However, cases increased rapidly in December 2020, with the outbreak peaking in August 2021, when 589,415 cases were reported. In Thailand, the COVID-19 preventive measures in Thailand were implemented from March 2020 to April 2022, specifically social distancing, stay at home, curfews, and a nationwide lockdown. The preventive measures have been loosened since May 2022, allowing schools and restaurants to reopen.

An acute undifferentiated febrile illness (AUFI) is an undifferentiated infectious febrile illness of short duration (<14 days). An AUFI is a common presentation of a tropical or bacterial infection that does not cause obvious local symptoms or target specific organs; the etiology varies widely, typically due to diverse pathogens, with regional variations. In Thailand, dengue fever was predominant, accounting for 11.8% of cases, followed by leptospirosis, typhoid fever, enteric fever, scrub typhus, influenza, and malaria.^2^^–^^5^ Because an AUFI does not show specific symptoms, it is difficult to diagnose based on clinical symptoms; epidemiological information can be the key to diagnosis.

Malaria, dengue fever, leptospirosis, scrub typhus, and typhoid fever are either insect-borne infections or food- and water-borne infections. Dengue fever is a mosquito-borne viral disease transmitted by *Aedes* mosquitoes. Thailand reported a marked decrease in dengue infections during the COVID-19 pandemic. Malaria is a mosquito-borne parasitic infection transmitted by *Anopheles* mosquitoes. Common symptoms of malaria include fever, chills, and flulike illness. Malaria incidence increased after the COVID-19 pandemic in Africa; however, the epidemiological characteristics of malaria in Southeast Asia are not well described. Leptospirosis infection occurs from exposure to water contaminated with animal urine or from contaminated food or water intake. Scrub typhus, a tick-borne rickettsiosis caused by *Orientia tsutsugamushi*, is endemic in Asia and Australia. Typhoid (enteric) fever is a food or waterborne infection caused by *Salmonella enterica* serovar Typhi (*S. typhi*). The case numbers of typhoid fever vary geographically, with south-central and Southeast Asia having the highest reports.

COVID-19 preventive measures, such as wearing masks, handwashing, and city lockdowns, decreased respiratory infections such as pneumonia and influenza in Japan and Thailand.^6^ These measures limited person-to-person contact through social distancing, mask-wearing, hand hygiene, and lockdowns, effectively reducing respiratory infections worldwide. Non-pharmaceutical interventions and behavioral changes have been shown to mitigate seasonal influenza as well.^7^ However, trends of mosquito-borne, tick-borne, food, or waterborne infections have not been well studied. The objective of this research was to determine the epidemiological characteristics of acute febrile disease in Thailand when infection control measures have been implemented during a global infectious disease outbreak such as COVID-19. Changes in this epidemiological feature are helpful in diagnosing AUFIs. In addition, it will be possible to make similar accurate predictions of infectious disease outbreaks in the future.

## MATERIALS AND METHODS

This retrospective descriptive study used national data from the period January 2018–December 2022 provided by the Ministry of Public Health and the Department of Disease Control in Thailand.^8^ We divided the timeline into three periods: phase 1 from January 2018 to February 2020 (before COVID-19), phase 2 from March 2020 to April 2022 (during the COVID-19 pandemic with preventive measures), and phase 3 from May to December 2022 (during the COVID-19 pandemic with loosening of preventive measures).

The study analyzed the annual case numbers of COVID-19, dengue, malaria, leptospirosis, scrub typhus and typhoid fever. The positive COVID-19 cases included hospital cases and high-risk contacts. Only cases diagnosed by reverse transcription polymerase chain reaction were reported as positive for COVID-19. Positive individuals were then isolated in quarantine sites for at least 14 days from the onset of symptoms to prevent transmission. The percentage increase or decrease was calculated by comparing the number of cases during these years to the mean number of cases. Geographic heat maps of reported cases (by province) and disease case numbers were created by using Excel 2021, with lighter colors representing lower values and darker shades standing for higher ones.

Statistical analysis included calculating the median and interquartile range (IQR). Characteristics were recategorized, and differences among phase 1 (before COVID-19), phase 2 (preventive measures), and phase 3 (loosened preventive measures) were compared using the Kruskal–Wallis test followed by the Steel–Dwass test. Statistical significance was defined as two-sided *P* <0.05. All statistical analyses were performed using SPSS for Windows v. 21.0 (IBM Corp,, Armonk, NY) This study used anonymized and deidentified data from the Thailand National database; therefore, ethical review was not required.

## RESULTS

**Figure 1.**
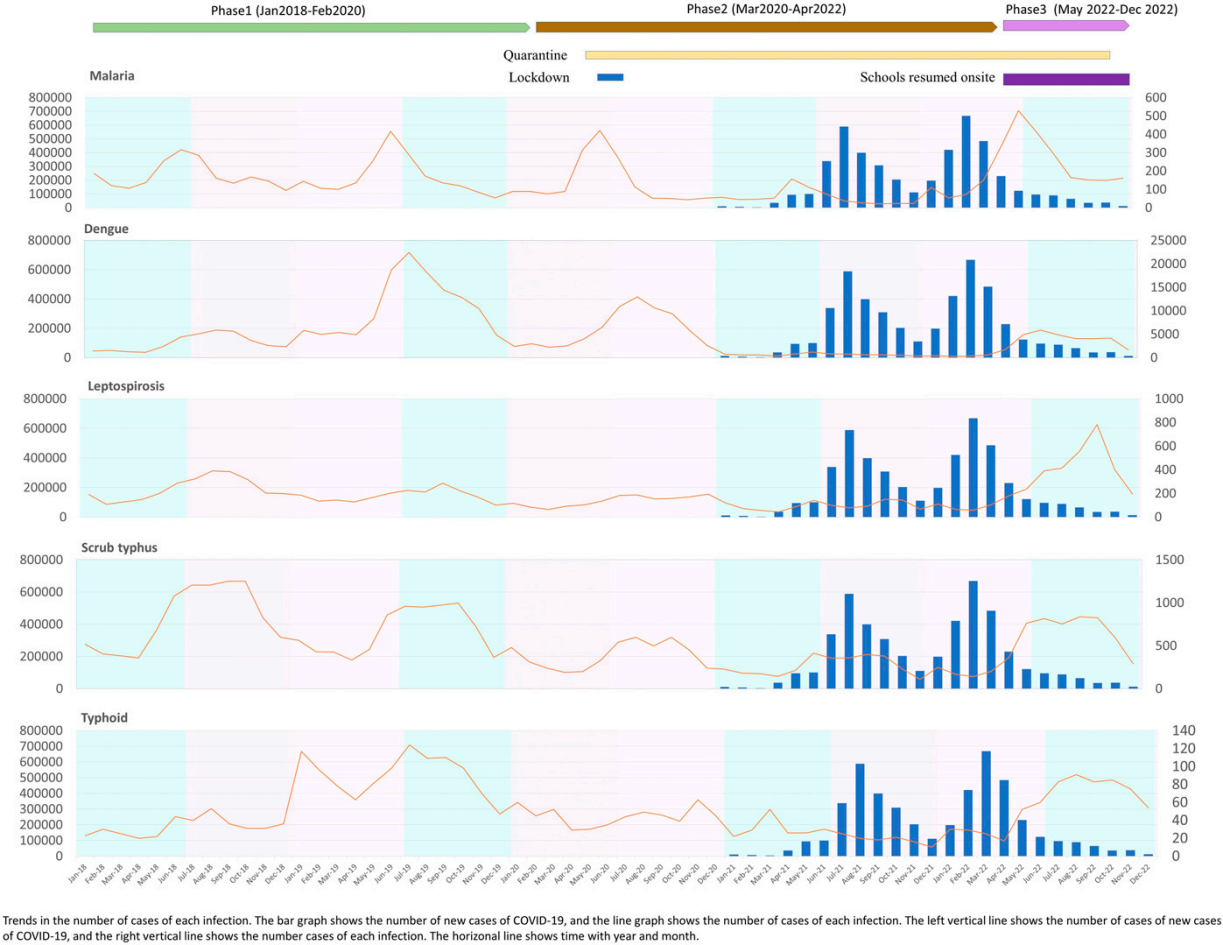
Trends of acute febrile diseases and COVID-19 in Thailand: 2018–2022.

COVID-19 was first reported in Thailand on January 12, 2020, and up until December 31, 2022, the total number of cases was 4,723,920 according to the data reported by the Ministry of Public Health of Thailand (Figure 1). The number of reported deaths due to COVID-19 during that period was 33,669. The number of COVID-19 cases was relatively constant from January 2020 to November 2020; the number of cases per day was less than 2,000. However, the number of patients increased rapidly in December 2020. The highest case number occurred in March 2022, at 668,088 cases per month.

**Figure 2.**
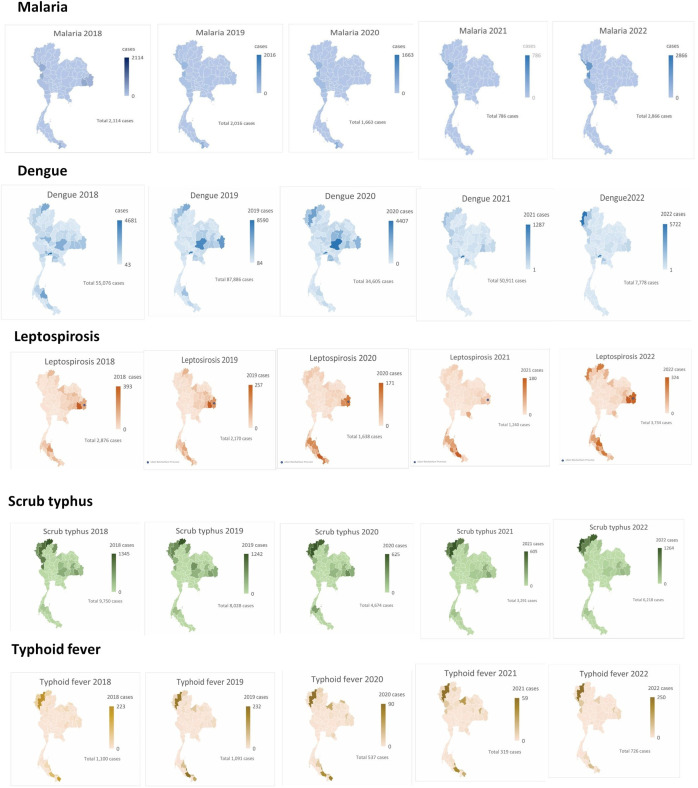
Geographic heat maps of acute febrile diseases in Thailand: 2018–2022.

The first wave of the COVID-19 outbreak began in March 2020, at which point the Thai government declared a state of emergency, imposed a national lockdown, and established a quarantine system. A national lockdown was enforced from May 2 to June 15, 2020, and a national curfew was enforced from April to June 2020. In May 2022, all schools in Thailand resumed on-site classes and relaxed their COVID-19 control policies, as shown in Figure 2.

The case numbers of five etiologies of AUFIs in Thailand (i.e., dengue, malaria, leptospirosis, scrub typhus, and typhoid fever) are shown in Table 1 and Figure 1. The case numbers of all diseases decreased during the period when preventive measures of the COVID-19 pandemic were loosened. The number of malaria cases decreased during phase 2 (Figure 1). From 2018 to 2022, a high number of malaria cases was reported in July in the rainy season, and fewer cases were reported in October 2021 in the winter season. The number of malaria cases increased again by 39% in 2022 (Table 1).

**Table 1:** Number of cases of each infection during the study period

AFI and COVID-19 Trends	2018	2019	2020	2021	2022	% Increase/Decrease in Cases in 2020	% Increase/Decrease in Cases in 2021	% Increase/Decrease in Cases in 2022
Malaria	2,114	2,016	1,663	786	2,866	−19%	−62%	39%*
Dengue	55,076	87,886	34,605	50,911	7,778	−52%	−29%	−89%
Leptospirosis	2,876	2,170	1,638	1,240	3,734	−35%	−51%	48%*
Scrub Typhus	9,750	8,028	4,674	3,291	6,218	−47%	−63%	−30%
Typhoid	1,100	1,091	537	319	726	−51%	−71%	−34%
COVID-19	–	–	–	2,200,707	2,260,485	–	–	3%*

COVID-10 = coronavirus disease 2019.

* The positive percentages indicate an increase in the number of cases.

The number of dengue cases decreased during preventive measures (Table 1). Similar seasonal trends were observed in 2019, 2020, and 2022, with an epidemic in the rainy season from June to October. After January 2021, a few dengue cases were reported, but from May 2022, the number of dengue cases started to increase. Epidemics occurred cyclically in the region every 1–2 years.

In addition, the number of leptospirosis cases officially reported in Thailand was 2,876 in 2018, 2,170 in 2019, 1,638 in 2020, 1,150 in 2021, and 3,482 in 2022. The disease was also epidemic in the rainy and winter seasons from June to October and decreased in winter from November to February. From May 2022, leptospirosis cases increased by 48% in 2022 (Table 1); the highest number of cases (781 cases) was reported on October 22.

The number of scrub typhus cases in Thailand from 2018 to 2022 is shown in Table 1. The scrub typhus season usually starts in April, and June and July are the peak months. The highest number of cases was reported in 2018. The number of scrub typhus cases also decreased after the loosening of preventive measures. Beginning in May 2022 (i.e., phase 3), the number of scrub typhus cases increased.

Typhoid fever cases from 2018 to 2022 are shown in Table 1. The highest number of cases was reported in 2019 at 1,091 cases, and the lowest was reported in 2021, at 295 cases. The seasonal trend is not obvious compared with the other AUFIs; the highest number was reported in winter, January 2019. Typhoid fever cases decreased during phase 2 and then increased again starting in May 2022 during phase 3.

Table 2 shows changes in reported cases during the study period. The results indicate that the case numbers of malaria and leptospirosis significantly decreased during phase 2 (*P =* 0.02) and increased again during phase 3 (*P =* 0.01). The median case numbers of malaria were 137 (IQR: 104–204) during phase 1, 55 (IQR: 44–111) during phase 2, and 120 (IQR: 154–395) during phase 3. Moreover, typhoid and scrub typhus case numbers showed a significant decrease during phase 2 (*P =* 0.02) and increased again during phase 3 (*P =* 0.01). The median case numbers of typhoid were 50 (IQR: 31–96) during phase 1, 29 (IQR: 21–44) during phase 2, and 79 (IQR: 55–84) in phase 3. The median case numbers of scrub typhus were 639 (IQR: 420–977) during phase 1, 239 (IQR: 188–401) during phase 2, and 759 (IQR: 418–822) during phase 3.

**Table 2:** Median and IQR of the acute febrile diseases in phase 1 (before COVID-19), phase 2 (COVID-19 with preventive measures), and phase 3 (COVID-19 with the loosening of preventive measures) and the comparison by each phase

Acute Febrile Illness	Phase 1	Phase 2	Phase 3	Phase 1 vs. Phase 2 *P*-Value	Phase 1 vs. Phase 3 *P*-Value	Phase 2 vs. Phase 3 *P*-Value
	Median (IQR)	Median (IQR)	Median (IQR)			
Malaria	137 (104–204)	55 (44–111)	120 (154–395)	0.01*	0.03*	0.02*
Dengue	4,958 (2,418–8,867)	778 (538–4,492)	4,120 (2,321–4,908)	0.02*	0.63	0.12
Leptospirosis	196 (133–240)	104 (70–150)	395 (206–519)	<0.01*	0.01*	0.01*
Scrub typhus	639 (420–977)	239 (188–401)	759 (418–822)	<0.01*	0.88	0.02*
Typhoid	50 (31–96)	29 (21–44)	79 (55–84)	0.02*	0.44	0.02*

COVID-10 = coronavirus disease 2019; IQR = interquartile range.

* *P* <0.05 was considered statistically significant.

## DISCUSSION

The common etiological pathogens of AUFIs in Thailand—malaria, dengue, leptospirosis, scrub typhus, and typhoid fever—decreased after the COVID-19 pandemic. However, for most of the AUFI etiologies, the numbers of cases increased again after May 2022. In May 2022, the Thailand government loosened its policies of preventive measures against COVID-19, and all schools in the country resumed on-site classes. We previously reported that respiratory and intestinal infections decreased during the COVID-19 pandemic,^9^ but in addition to that, mosquito-borne infections such as malaria, dengue disease, tick-borne infections such as scrub typhus, and zoonotic waterborne diseases such as leptospirosis decreased during COVID-19 and increased again at the time of loosening of COVID-19 preventive measures.

The increase after the loosening of COVID-19 preventive measures can be attributed to several factors, including lifting of restrictions, which led to increased travel, outdoor work, and social gatherings, heightening exposure to disease-prone environments. With relaxed measures, people were less vigilant about hygiene practices, contributing to infection resurgence. Lockdowns meant limited interactions with pathogen-rich environments, but after loosening, exposure to vectors such as mosquitoes and contaminated water increased. The pandemic diverted healthcare resources, disrupting routine public health programs and facilitating infection resurgence. Increased mobility and travel after the pandemic allowed wider pathogen dissemination. In addition, factors such as increased rainfall created favorable conditions for vector breeding, boosting infection rates.

However, there was a decrease in the number of malaria cases during the preventive measures period, and then it increased again after May 2022. Globally, there were an estimated 241 million malaria cases in 2020 in 85 malaria-endemic countries, increasing from 227 million in 2019.^8^ Most malaria cases were reported in the African region. The present study found a decrease in malaria cases during the COVID-19 pandemic, consistent with the WHO Southeast Asia Region; malaria cases decreased by 78%, from 23 million in 2000 to about 5 million in 2020.^10^ Malaria remains a public health priority in Thailand, with more than 13 million people, about 19% of the total population, currently at risk. Between 2016 and 2017, Thailand’s malaria cases decreased by 39%.^11^^,^^12^ Owing to elimination efforts, transmission has been reduced in and around forested areas along international borders.^13^ Thailand has three principal malaria-endemic regions: the northeast, bordering Lao People’s Democratic Republic; the west, Myanmar; and the south, bordering Malaysia.^14^ After COVID-19 began in 2020, the case numbers of malaria dropped rapidly, starting on August 2020. This result suggests that COVID-19 lockdowns, such as the change in lifestyle, social lockdown, and immigration restrictions, decreased malaria transmission in Thailand. This result is similar to that seen in the reduction in malaria cases in Peru was 20% from February to March 2020; 44% from March to April; 88% from April to May 2021; and 99% from May to June 2021.^15^^,^^16^ In Thailand, there was a high number of malaria cases in July in the rainy season, and Tak Province, Kanchanaburi Province, and Ratchaburi Province, at the Thai Myanmar border, were the three areas with the highest case numbers in Thailand. They have historically recorded some of the highest numbers of malaria cases across the country. They reported the highest number of malaria cases nationwide in 2018 (335 cases), 2019 (380 cases), 2020 (294), and 2021 (200 cases), as shown in Figure 2.

These findings suggest that in 2020, when COVID-19 lockdowns started, the migration of people between the two areas was restricted and may have contributed to decreased transmission in the areas. On the other hand, in 2022, when COVID-19 restrictions were fully relaxed, the number of malaria cases increased rapidly. This pattern could be the result of multiple factors. Human population movement has frequently been cited as a major factor in the persistence of malaria along this international border. Migrants from a malaria-endemic region can bring the parasite with them. Some studies have indicated a higher prevalence of malaria in migrants from Myanmar, leading to the suggestion that migrants are a major public health problem for Thailand.

Dengue cases decreased in Thailand during the COVID-19 lockdown. Dengue typically occurs every 2–3 years, and staying at home may have reduced exposure to mosquitoes, particularly during the daytime in urban settings. In the present study, the effect of COVID-19–related social distancing interventions were examined. It was found that dengue cases decreased in Thailand in 2020 and 2021, with this decrease being especially marked in 2021. The decrease in the number of cases is consistent with a previous report in South America; at a national level, increasing reports of COVID-19 interestingly contrasted with fewer reported dengue fever cases.^17^ However, a positive relationship was found between monthly dengue cases and COVID-19 cases in Vietnam and Indonesia.^18^ There are many factors related to the dengue case numbers. A study in India found that the distribution density of immature individuals of *Aedes* mosquitos increased drastically during the COVID-19 lockdown owing to paused vector control programs.^19^^,^^20^ During the COVID-19 lockdown, Thailand experienced its lowest number of reported dengue cases compared with the 3-year average with the government health system. The dengue outbreak control program conducted by the government was interrupted during the lockdown. The public health staff and the military were heavily involved with COVID-19 mitigation activities, with less emphasis on dengue source reduction. However, community engagement to actively remove breeding habitats in and around homes may have improved during an extended period spent at home during the lockdown. People had more time to pay attention to vector breeding on their premises. Moreover, after the number of COVID-19 pandemic dengue cases started to increase in June 2022, during the rainy season, dengue cases increased in the central region and in Mae Hong Son, the northern part of Thailand, as shown in Figure 1.^21^ However, a previous study suggested that the highest dengue incidence rates were reported in children (10–14 years of age) and young adults (15–24 years of age).^22^ Schools have previously been suggested as potential sites for dengue transmission based on adult vector and larvae surveys, but results from many studies have been inconsistent.^23^ Primary school students were considered a vulnerable group because of the lack of protection from and prevention of mosquito bites. Previous research highlights that children are especially susceptible to diseases like dengue and Zika, which can lead to severe health outcomes. Public health initiatives, including the use of permethrin-impregnated school uniforms, have been explored to enhance protection for students.^24^ The present study found that dengue cases started to increase after the COVID-19 pandemic, perhaps because schools reopened after the COVID-19 pandemic.

Leptospirosis was first reported in Thailand in 1942. The number of reported leptospirosis cases increased from 398 in 1996 to 14,285 in 2000.^25^ From 2003 to 2012, there were a total of 41,089 cases of leptospirosis reported to the Bureau of Epidemiology, Ministry of Public Health, Thailand. From 2018 to 2021, during the COVID-19 pandemic, a total of 15,668 leptospirosis cases were reported. The number of leptospirosis cases decreased in 2020 and 2021. This report was inconsistent with the previous report; in Sri Lanka, the number of leptospirosis cases increased in the second quarter of the COVID-19 pandemic.^26^ The occurrence of human leptospirosis is associated with agricultural work and outdoor occupations related to animals and environmental water, and water consumption is a significant risk factor for human leptospirosis.^27^^,^^28^ The number of reported leptospirosis cases decreased during the COVID-19 pandemic owing to several factors associated with preventive measures and lifestyle changes. These reasons included reduced outdoor activities, as lockdowns and stay-at-home orders decreased activities such as farming, fishing, and water-related work, which are common leptospirosis risk factors. Travel restrictions limited movement between regions and countries, reducing exposure to rural and agricultural areas where leptospirosis is prevalent. Improved hygiene practices, with an emphasis on hand hygiene, disinfection, and general sanitation to prevent COVID-19, also reduced leptospirosis transmission through contact with contaminated water or soil. Quarantine measures, such as isolating potentially infected individuals, helped prevent community spread of leptospirosis. Decreased human-animal interaction due to lockdowns and reduced mobility led to less contact with animals such as rodents and livestock, lowering infection risks.

Leptospirosis is a major health problem in Thailand, particularly in northeast Thailand. The northeastern region of Thailand had the highest burden of leptospirosis compared with the other regions. The highest case report number was found in Si Sa Ket, a province in northeast Thailand, followed by Ubon Ratchathani. In Si Sa Ket province, the numbers of leptospirosis cases in 2018, 2019, 2020, 2021, and 2022 were 393, 257, 108, 48, and 324 cases per year, respectively. Si Sa Ket is known to experience outbreaks of leptospirosis throughout the year, especially during the rainy season, as shown in Figure 2. This is consistent with previous studies.^29^ Important problems of managing leptospirosis were found to be that people in this area lacked knowledge of the disease, including the need to protect themselves from infection by wearing boots or gloves while working at agricultural activities. A study from Sri Lanka reported that high-risk occupations, mostly those related to agricultural work, were associated with leptospirosis.^30^ However, the epidemic season of leptospirosis in the northeastern region of Thailand is the rainy season. From September to October 2022, the amount of rainfall increased around the Ubon Ratchathani and Si Sa Ket areas. During heavy rain, when flooding increases, *Leptospira* spp. can more easily contaminate the environment. Previous studies pointed out that working or living in flooded areas has been identified as a significant factor for increasing leptospirosis infection.^31^ Flooding was also observed to be an important risk factor in other countries such as Brazil^32^ and Malaysia.^33^

Scrub typhus is another AUFI caused by bacterial infections. It is a zoonotic tick-borne disease caused by the gram-negative, obligate intracellular bacillus *Orientia tsutsugamushi*. It is transmitted via bites of the chigger (larva of trombiculid mites). Scrub typhus in Thailand has increased significantly over the last two decades, from 2003 to 2018, before the COVID-19 pandemic.^34^ The present study demonstrated that the number of scrub typhus cases decreased after the COVID-19 pandemic in 2020 and 2021. This result is consistent with a report from Nepal; scrub typhus cases decreased in 2020.^35^ In Thailand, the disease burden was highest in working-age persons.^33^ Scrub typhus was more common in older age groups in the other five countries, particularly Japan and South Korea, related to farming communities.^36^^,^^37^ Many factors affected the number of cases of scrub typhus. Of these, the environment, such as temperature and humidity, could have affected the number and distribution of chiggers and rodents.^38^ During a social distancing lockdown, staying at home may reduce the risk of contact with rodents. In Thailand, the disease case numbers reported peaked in July, with a smaller peak in November. Chiang Rai province is the northernmost province of Thailand with the highest number of scrub typhus cases reported in Thailand (Figure 2). In June–July in the northern part of Thailand, agricultural activity, such as the clearing of scrubs or new vegetation, starts. A previous study reported that this habitat was associated with the presence of infected rodents and chigger mites as important ecological factors.^33^ However, since the COVID-19 pandemic began, the number of case reports has decreased, which is consistent with previous reports from Taiwan. A recent study from Taiwan reported that the incidence of scrub typhus in 2021, when national lockdown measures and travel restrictions were implemented, was reduced to <50% of the average incidence over the past 5 years.^39^

Similar persistence patterns were also observed for typhoid fever, or enteric fever, which is an endemic disease in Thailand.^40^ In 2017, an estimated 14 million cases of enteric fever occurred worldwide, resulting in about 136,000 deaths. More than 80% of these cases occurred in South and Southeast Asia and sub-Saharan Africa.^41^ Typhoid fever cases decreased during the preventive mature period. The highest number of cases was reported in 2018 (1,100 cases), and the lowest number of cases was reported in 2021 (319 cases) (Figure 1). Because the transmission of typhoid fever is via food or waterborne sources,^42^ it was expected that the number of cases might drop during the COVID-19 pandemic due to the preventive measures implemented. These measures included travel restrictions that limited movement and exposure to contaminated food or water sources, quarantine measures that isolated exposed individuals, preventing the spread within communities, and stay-at-home orders and lockdowns that reduced public gatherings and dining out, thus minimizing exposure to contaminated food and water. Enhanced hygiene practices emphasized hand hygiene and sanitation, likely reducing fecal-oral transmission routes. School and business closures, including closing schools, restaurants, and other businesses, also reduced the chances of typhoid outbreaks. These combined interventions disrupted the typical transmission routes of typhoid fever, leading to a significant decrease in reported cases during the pandemic. This result was consistent with a report in the Netherlands, where the transmission of salmonellosis decreased during the COVID-19 pandemic.^43^ In addition, imported cases of typhoid may have decreased owing to the decrease in the number of foreign visitors. The symptoms of typhoid fever are similar to the clinical presentation of COVID-19. Therefore, the similarities in the clinical presentations of these two diseases could lead to misdiagnosis. The diagnostic delay due to symptom similarity of COVID-19 with other infectious diseases has also been reported in other countries.^44^

The present study has several limitations. First, the number of patients after the COVID-19 pandemic may have been underestimated. Patients might not have visited hospitals because of the fear of doing so during the COVID-19 pandemic. Second, during the COVID-19 pandemic period, the number of foreign tourists dropped, which may have contributed to the reduction of cases. In addition, it is believed that a number of unmeasured confounding factors were present and may have affected the number of cases of infection. Third, the lack of detailed cases reported and missing case numbers available to us may have affected the accuracy of the AUFI cases in Thailand.

To address these limitations, future research should incorporate more comprehensive data collection methods, including active surveillance and longitudinal studies. Investigating the impact of specific preventive measures in controlled settings could help establish clearer causal relationships. In addition, examining the role of environmental factors in disease transmission would provide a more nuanced understanding of their influence on public health outcomes.

In conclusion, this study investigated the impact of COVID-19 and its preventive measures on the case numbers of AUFIs in Thailand, utilizing national data from 2018 to 2022. The study showed that implementing COVID-19 preventive measures significantly decreased the etiological pathogens of AUFIs during phase 2, such as dengue, malaria, leptospirosis, scrub typhus, and typhoid fever. However, with the loosening of these measures in 2022, there was a notable resurgence in malaria and leptospirosis cases, increasing by 39% and 48%, respectively. In addition, AUFI etiologies need to be reevaluated in hospital settings after the COVID-19 pandemic; the proportions and etiological organisms may have changed significantly from previous studies. This research not only underscores the importance of considering these dynamics in the differential diagnosis of AUFIs but also aids in predicting the future landscape of major infectious diseases in the post–COVID-19 era. Continuous monitoring and reevaluation of AUFI etiologies in hospital settings are essential for adapting to evolving epidemiological patterns and effectively informing public health strategies.
